# Autologous Stem Cell Transplantation in Multiple Myeloma: Where Are We and Where Do We Want to Go?

**DOI:** 10.3390/cells11040606

**Published:** 2022-02-10

**Authors:** Sonia Morè, Laura Corvatta, Valentina Maria Manieri, Francesco Saraceni, Ilaria Scortechini, Giorgia Mancini, Alessandro Fiorentini, Attilio Olivieri, Massimo Offidani

**Affiliations:** 1 Clinica di Ematologia, Azienda Ospedaliero-Universitaria Ospedali Riuniti di Ancona, 60126 Torrette, Italy; sonia.more@live.it (S.M.); valentina.manieri95@gmail.com (V.M.M.); francesco.saraceni@ospedaliriuniti.marche.it (F.S.); ilaria.scortechini@ospedaliriuniti.marche.it (I.S.); giorgia.mancini@ospedaliriuniti.marche.it (G.M.); alessandro.fiorentini2@ospedaliriuniti.marche.it (A.F.); a.olivieri@univpm.it (A.O.); 2 U.O.C. Medicina, Ospedale Engles Profili, 60044 Fabriano, Italy; laura.corvatta@sanita.marche.it

**Keywords:** multiple myeloma, autologous stem cell transplantation, induction, consolidation, maintenance

## Abstract

The introduction of high-dose therapy in the 1990s as well as the development of drugs such as thalidomide, lenalidomide, and bortezomib in the 2000s led to an impressive improvement in outcome of patients with multiple myeloma (MM) eligible for autologous stem cell transplantation (ASCT). Clinical trials conducted in the first ten years of the twenty-first century established as standard therapy for these patients a therapeutic approach including induction, single or double ASCT, consolidation, and maintenance therapy. More recently, incorporating second-generation proteasome inhibitors carfilzomib and monoclonal antibody daratumumab into each phase of treatment significantly improved the efficacy of ASCT in terms of measurable residual disease (MRD) negativity, Progression Free Survival (PFS), and Overall Survival (OS). The availability of techniques such as multiparameter flow cytometry (MFC) and next-generation sequencing (NGS) for MRD assessment allowed the design of MRD-based response-adjusted trials that will define, in particular, the role of consolidation and maintenance therapies. In this review, we will provide an overview of the most recent evidence and the future prospects of ASCT in MM patients.

## 1. Introduction

Multiple myeloma (MM), a clonal plasma cells hematologic malignancy, accounts globally for 0.9% of all cancers diagnosed; the incidence increased by 126% from 1990 to 2016, especially in the developed world, e.g., Australia, western Europe, and US, where in 2020 it accounted for 1.8% of all cancers. Median age at diagnosis is 69 years, and about 65% of patients with newly diagnosed MM (NDMM) are between 45 and 74 years old [1]. MM is characterized by extreme inter-and intra-patient heterogenicities so much that there is the idea that MM should no longer considered a single disease but as different entities that are increasingly shared. MM is still an incurable disease, but this does not mean that there have been no changes in the MM outcome over the years since with the introduction of high-dose therapy in the 1990s and novel agents in the 2000s, a substantial improvement in overall survival (OS) has been observed, particularly in younger patients [2,3]. A recent analysis of 4329 NDMM patients treated with autologous stem cell transplantation (ASCT) at the University of Arkansas from 1989 through 2018 demonstrated the possibility of curing a fraction of patients, ranging from 6.3% to 31.3%, depending on the year of treatment, with many patients who achieved normal life expectancies [4]. Another large series of patients treated in France in “real-life” studies confirmed these results, reporting a median OS longer than 10 years in patients who underwent ASCT between 2010 and 2014 [5]. The ability of extending long-term survival has to be attributed to a deeper degree of response obtained by incorporating novel agents into the transplantation strategy [4]. On the other hand, a prospective study (BMTSS) in which 1906 patients who underwent ASCT were followed for a median of nine years showed a decrease in late mortality related to myeloma, infection, and cardiac events over the past 25 years [6]. In transplant-eligible (TE) NDMM patients, ASCT remains the standard of care recommended by international guidelines such as by ASCO [7] and ESMO [8] since a significant benefit of ASCT vs. conventional therapy was demonstrated, not only in patients receiving “old” chemotherapies in the control arm [9,10,11,12,13,14], but also in those treated with new triplet combinations including proteasome inhibitors (PIs) and immunomodulatory agents (IMiDs) [15,16,17,18,19]. 

However, the increased efficacy of triplet or quadruplet induction regimens in achieving MRD negativity raised the question regarding the role of early ASCT, and several clinical trials are assessing this issue. Although the current transplantation approach consists of various phases such as induction, ASCT, consolidation, and maintenance, it remains to be clarified the real importance of consolidation therapy as well as the best maintenance therapy and how long it should last. The concept of tailoring therapy based on MRD status instead of prolonging potentially toxic and unnecessary therapies is explored in many ongoing studies, and personalized therapy will be probably the future of MM therapy. The treatment of high-risk (HR) MM patients remains a challenge since the definition of HR disease is controversial and the optimal choice for it is not well defined. In this review, we will summarize the main fixed points about ASCT in MM, highlighting the most recent data concerning tailored therapies and HR MM treatment.

## 2. Where We Are with Induction Therapy

The addition of bortezomib, the first-in-class proteasome inhibitor (PI) that reversibly inhibits the proteasome causing myeloma cells’ apoptosis to the previously used doublets has been one of the greatest breakthroughs in MM treatment. The combination of bortezomib, dexamethasone, and the immunomodulatory drug thalidomide (VTD) proved to be effective in various studies. In the phase III GIMEMA-MMY-3006 trial, comparing VTD vs. TD as induction (three cycles) and consolidation (two cycles) after two rounds of ASCT, patients who received induction with VTD had better responses than those who received TD only (≥ PR: 93% vs. 79%, *p* < 0.0001; ≥ VGPR: 62% vs. 28%, *p* < 0.0001), translating into a significantly better PFS (HR = 0.62, *p* < 0.0001) [20]. The superiority of VTD compared to TD was confirmed by another phase III study by PETHEMA/GEM, comparing VTD vs. TD vs. VBMCP/VBAD/B, and showing better outcomes in terms of CR rates (35% vs. 14%, *p* = 0.001) and median PFS (56.2 months vs. 28.2 months, *p* = 0.01) in patients receiving VTD vs. TD for six cycles as induction therapy before ASCT [21]. The IFM2013-04 trial showed the superiority of the VTD arm compared with bortezomib, cyclophosphamide, and dexamethasone (VCD) arms in terms of ORR (92.3% vs. 83.4%), even though it did not find any significant difference in terms of CR rates [22]. Adding cyclophosphamide to the triplet VTD did not result in a higher response rate, with 51% and 44% of patients achieving nCR/CR in the VTD and VTDC arm after induction, respectively [23]. Due to these results, especially in Europe, VTD has been considered the standard care for induction therapy in transplant-eligible NDMM [24].

Lenalidomide, a second-generation IMID, was used instead of thalidomide in combination with bortezomib and dexamethasone (VRd) and compared with Rd for the first time in the phase III SWOG S0777 trial, including patients of all ages and without intent of immediate ASCT. After a median follow-up of 84 months, median PFS, the primary endpoint of the study, was 41 months for VRd and 29 months for Rd (*p* = 0.003); OS was not reached in the first arm and was reached at 69 months in the second one [25]. Three cycles of VRD as induction were used in the phase III IFM 2009 study [17], whereas in the PETHEMA/GEM2012 study, patients received VRD for six cycles followed by ASCT conditioned with busulfan plus melphalan vs. melphalan. After induction, 83.4% of patients achieved at least PR, 66.6% at least VGPR, and 33.4% CR, and 28.8% patients obtained MRD negativity at a level of 3 × 10^−6^ using next-generation flow (NGF) [26].

The VRD regimen has become another standard of care for newly diagnosed TE MM patients, and it is mainly used in the U.S. In a study including the largest cohort of patients treated with VRD in the U.S. (1000 patients among whom 751 received up-front ASCT), after four cycles of induction, ORR was 97%, ≥ VGPR 67.6%, and CR 35.9%. After a median follow-up of 102 months, median PFS and OS in patients who underwent ASCT were 63 months and 123.4 months, respectively [27]. Another “real-life” experience reported similar data since the median PFS was 50 months and the median OS was 101.7 months in patients treated with 4–6 VRD cycles and ASCT [28]. However, no prospective randomized trial compared VTD to VRD although an integrated retrospective analysis of randomized trials reported a significantly higher ≥ VGPR rate after six cycles of VRD compared to VTD (70% vs. 60%, respectively) with a similar safety profile [29].

Although the phase III ENDURANCE trial that enrolled patients without ASCT intention did not demonstrate superiority of KRd over VRd [30], a triplet including carfilzomib, a second-generation PI with a peculiar mechanism of action compared with bortezomib, was found to induce high responses when used as induction therapy before ASCT [31,32]. 

In the phase II FORTE trial, aiming to explore different carfilzomib-based induction and consolidation therapies with or without ASCT, patients randomized to receive four cycles of carfilzomib, lenalidomide, and dexamethasone (KRd) as induction had significantly higher ≥ VGPR, the primary endpoint, compared to patients treated with four cycles of carfilzomib, cyclophosphamide, and dexamethasone (KCd) (70% vs. 53%, OR = 2.14, *p* = 0.0002) [19].

The landscape of MM treatment has once more been changed with the introduction of monoclonal antibodies (mAb) such as daratumumab, and several clinical studies assessed the impact of adding a mAb to the afore-mentioned triplets. In the phase III CASSIOPEIA trial, comparing four cycles of VTd vs. VTd plus daratumumab (D-VTd) as induction before ASCT, the quadruplet was found to induce at least VGPR in 65% of patients vs. 56% in those treated with VTd [33]. No randomized trial directly compared D-VTd with VRd. Adding daratumumab to the VRd combination (D-VRd) resulted in high-quality responses as reported by the phase II GRIFFIN study in which after four cycles of D-VRd, at least VGPR was 72% and MRD negativity was 21.2% compared to 56.7% and 5.8%, respectively, for VRd [34]. The phase III PERSEUS trial is evaluating the efficacy and safety of D-VRd (with subcutaneous daratumumab) vs. VRd. After four cycles of daratumumab combined with KRd, 90% of patients achieved VGPR, with 38% MRD negativity at a level of 10^−5^ and 24% at a level of 10^−6^ in the phase II MASTER study [35]. This rate of high-quality response with D-KRd has been reported in the single-center nonrandomized MANHATTAN trial in which eight courses of D-KRd (weekly carfilzomib) led to an ORR of 100%, with 95% of patients obtaining at least VGPR and 71% MRD negativity. Patient candidates for ASCT were offered stem cells after 4–6 cycles of D-KRd [36]. By using oral PI ixazomib in combination with lenalidomide, dexamethasone, and daratumumab (IRd-Dara) as induction prior to ASCT, at least VGPR was achieved in 78% of patients after six cycles in the IFM 2018-01 study, with an MRD negativity of 28% at a level of 10^−5^ [37]. The phase II EMN 18 study is assessing the efficacy of daratumumab plus VCD (Dara-VCd) vs. VTd combinations administered as induction and consolidation after ASCT. 

**Figure 1:**
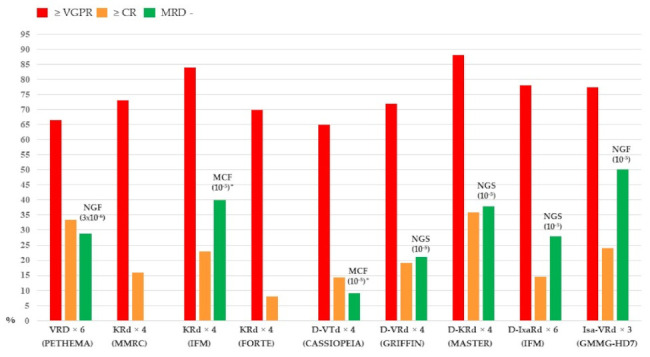
Response rates after induction therapy of the main triplet and quadruplet combinations evaluated in transplant-eligible newly diagnosed MM. VRD: bortezomib, lenalidomide, dexamethasone; KRd: carfilzomib, lenalidomide, dexamethasone; D-VTd: daratumumab, bortezomib, thalidomide, dexamethasone; D-VRd: daratumumab, bortezomib, lenalidomide, dexamethasone; D-KRd: daratumumab, carfilzomib, lenalidomide, dexamethasone; D-IxaRd: daratumumab, ixazomib, lenalidomide, dexamethasone; Isa-VRd: isatuximab, bortezomib, lenalidomide, dexamethasone * Only patients with ≥ CR.

Isatuximab, another mAb targeting a specific epitope on CD38, has been explored in the upfront setting after being approved in combination with dexamethasone and pomalidomide or carfilzomib in RRMM. The phase III GMMG-HD7 study is the first one randomizing patients to receive VRd or isatuximab plus VRd (Isa-VRd) in patients eligible for ASCT. The trial met its primary endpoint, an MRD negativity rate after induction therapy, since it was 50.1% vs. 35.6% in patients treated with Isa-VRd and VRd, respectively (OR = 1.83, *p* < 0.001), with at least 77.3% vs. 60.5% of respective patients achieving at least VGPR after three cycles [38]. As of earlier this year, the phase III EMN24 IsKia trial has started enrolling patients who are being randomized to receive either KRD or KRD plus isatuximab followed by ASCT and consolidation treatment. The benefit of adding mAb to standard therapy has not been shown with elotuzumab, targeting SLAMF7, as reported by the phase III GMMG-HD6 trial in which four cycles with Elo-VRd did not significantly increase a high-quality response after induction compared to VRd alone (≥VGPR = 58.6% vs. 52.9% for Elo-VRd and VRd, respectively; *p* = 0.14) [39]. Ultimately, with regard to induction therapy in patients who are eligible for ASCT, triplet, and even more so, quadruplet combinations including mAbs are able to induce high-quality responses with a significant proportion of patients achieving MRD negativity at the level of 10^−5^ (Figure 1). The unresolved question remains whether extending induction therapy beyond 4–6 cycles could lead to even higher responses, thus omitting ASCT altogether. 

## 3. Considerations with Stem Cell Mobilization and Harvesting

Mobilization is a crucial step in order to successfully harvest and collect the minimum target required to perform an autologous stem cell transplant (≥2 × 10^6^ cells/kg CD34+ peripheral blood stem cells), usually achieved by using granulocyte colony-stimulating factor (G-CSF) [40]. Because around 20% of patients fail to mobilize the adequate number of CD34+ stem cells, a combination of intermediate dose (ID)–cyclophosphamide (4 g/m^2^) and G-CSF (5 mg/kg/day started on day +2) has been established over the years as the standard mobilization regimen for those patients [40].

Plerixafor, a selective reversible inhibitor of the CXCR4 chemokine receptor, can be used as salvage therapy for patients who fail to mobilize. 

A risk-adapted use of plerixafor was explored in the study led by Prakash, where the patients whose CD34+ count on day 4 was < 20 cells/µl received plerixafor, resulting in a higher percentage of patients who reached the target of 2 × 106/Kg CD34+ than in the population who used plerixafor (96.2% vs. 87.2%) [41]. When added directly to G-CSF, plerixafor increases the number of patients meeting the collection goals, but it is not commonly used upfront due to the high cost of the drug. However, by reducing the median number of apheresis days (1.5 vs. 1 day, *p* < 0.001) and increasing the median number of CD34+ cells collected (6.6 vs. 8.5 × 106 cells/kg, *p* < 0.001), it actually reduces apheresis costs and results in net savings [42].

Cyclophosphamide use in mobilization can lead to febrile neutropenia, the need of blood transfusions, and hospitalizations. Because of this, chemo-free regimens have been tested.

The Italian study led by Lazslo et al. compared a prospective single arm of 20 patients mobilized with G-CSF plus plerixafor on demand with a retrospective control arm of 30 patients mobilized with ID–cyclophosphamide (4 g/m^2^) and G-CSF. The results showed that both groups eventually collected the minimum target required to perform a tandem transplant (≥4 million CD34+/Kg), even though the total average CD34+ yield was significantly higher in patients mobilized with cyclophosphamide and G-CSF (mean collection 10.6 × 106 cells/kg vs. 5.8 × 106 cells/kg; *p* = 0.004) [43]. In another study conducted at the Mayo Clinic, patients receiving chemo-mobilization had higher stem cell yields than the growth-factor-only cohort (median, 10.7 × 106 cells/kg vs. 8.77 × 106 cells/kg, respectively; *p* < 0.001), with no differences in terms of length of hospitalization, incidence of bacteremia, and days to engraftment [44].

Therefore, the role of chemo-free regimens is still to be determined. 

The current standard practice for ASCT in MM is to cryopreserve mobilized peripheral blood stem cells (PBSC) to be subsequently infused after planned high-dose chemotherapy. A possible alternative that is being increasingly explored is to perform ASCT without cryopreservation. The major advantage of such an approach is the possibility to allow transplant procedures in developing countries with limited access to facilities for stem cell cryopreservation. Indeed, the cost of cryopreservation contributes to approximately 15% of transplant costs, and using fresh cells could be the only chance for hematology departments without cryopreservation laboratories. Further, some recent evidence suggests faster engraftment with the non-cryo approach. Overall, there is lack of comparative studies of fresh vs. cryopreserved stem cells for ASCT in MM, and only limited retrospective data are available. Sarmiento et al. [45] designed a comparative study between the two techniques, and they did not find any difference in terms of apheresis products and their viability. Engraftment was significantly faster in the fresh cell group in which febrile neutropenia and severe mucositis rates were lower. In addition, length of hospitalization was five days shorter in the fresh cell group. There were no differences in terms of outcomes between the two groups. Joseph et al. [46] reported results from a retrospective analysis of 32 patients receiving fresh stem cell infusion and 32 patients receiving frozen ones (ASH 2020). Time to platelet engraftment and length of stay in the hospital were significantly shorter in the group receiving fresh cells. Time to engraftment and duration of neutropenia were not significantly different, despite earlier development of neutropenia that was observed in the fresh cell group. The CD34 cell dose was lower in the fresh cell group. The use of fresh cells also helped investigators to discharge patients early from the transplant clinic. Kulkarni et al. [47] performed a retrospective analysis on 224 MM patients who underwent ASCT with G-CSF-mobilized fresh cells. After apheresis, peripheral stem cells were stored at 4 °C in a blood bank refrigerator for up to 72 h. The median time to engraftment was 12 days (range 9–22) for neutrophil and 17 days for platelets (range 10–44), resulting in the use of this technique been adequate for ASCT in MM patients; 72 h is commonly considered the time limit not to exceed in order to keep an acceptable stem cell viability. However this threshold has not been clinically validated; recent evidence showed delayed engraftment in patients with lymphoma autografted with non-cryopreserved stem cells infused up to six days after collection [48]. Further, it should be highlighted that infusion of autologous fresh stem cells needs very precise planning of patients’ hospital stay, conditioning, and reinfusion, requiring an efficient program coordination. In fact, cryopreservation is necessary in certain scenarios wherein the transplant has to be delayed until after stem cell harvest or cells have to be stored for two transplants because a second mobilization is necessary if a second transplant is planned at relapse. Finally, a possible advantage of such approach is to avoid the consequences of dimethyl sulfoxide toxicity; nevertheless, the development of automatic washing methods led to significantly decreased adverse effects related to infusion without affecting stem cell counts or viability [49]. Several clinical studies have shown that autologous transplantation is feasible without cryopreserving the graft [45,46,49,50,51,52,53]. 

## 4. Where We Are with Consolidation Therapy Post-ASCT

**Figure 2:**
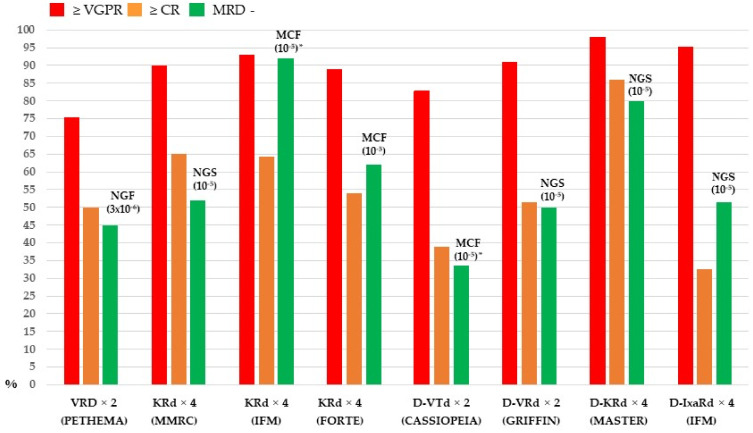
Response rates after consolidation therapy of the main triplet and quadruplet combinations evaluated in transplant-eligible newly diagnosed MM. VRD: bortezomib, lenalidomide, dexamethasone; KRd: carfilzomib, lenalidomide, dexamethasone; D-VTd: daratumumab, bortezomib, thalidomide, dexamethasone; D-VRd: daratumumab, bortezomib, lenalidomide, dexamethasone; D-KRd: daratumumab, carfilzomib, lenalidomide, dexamethasone; D-IxaRd: daratumumab, ixazomib, lenalidomide, dexamethasone * Only patients with ≥ CR.

Several recent studies confirmed the role of consolidation therapy in improving the response achieved after ASCT. In the phase III GIMEMA-MMY-3006 trial, after a median follow-up of 124 months, median PFS was 60 months in patients receiving VTD as induction and consolidation after two rounds of ASCT vs. 41 months in those treated with TD (*p* < 0.0001) whereas OS at 10 years was 60% and 46%, respectively (HR = 0.68, *p* = 0.0068). Notably, two cycles of VTD as consolidation were able to increase CR from 49% to 61%, whereas this increase was less significant for TD (from 40% to 47%) [54]. Achieving CR after ASCT has been correlated with longer PFS and OS [55], but the increased frequency and extent of response obtained with new regimens has led to the development and introduction of more sensitive tools for response assessment, such as measurable residual disease (MRD), whose use after treatment was found to better predict outcomes compared with conventional CR [56]. Moreover, MRD negativity was associated with a survival benefit regardless of the method used to detect it, i.e., next-generation flow cytometry (NGF) or next-generation sequencing (NGS) used to detect MRD. However, a minimum sensitivity of 10^−5^ is required, and sensitivity thresholds of methods are able to impact PFS (but also OS) with HRs for PFS improvements being 0.31 at a level of 10^−5^ and 0.22 at a level of 10^−6^ [57]. Clinical studies exploring triplets or quadruplets as consolidation therapy after ASCT demonstrated the possibility of achieving MRD negativity at a level of 10^−5^ in a substantial percentage of patients as shown in Figure 2. 

In the PETHEMA/GEM2012 study, patients (45%) MRD negative (with a median limit of detection of 3 × 10^−6^) after two VRD cycles as consolidation had a 3-year PFS of 87% vs. 50% in those with persistent MRD (HR 0.21, *p* < 0.001), with no significant difference between patients with standard vs. high risk cytogenetics [58]. To avoid bone marrow aspiration, in the same study, MRD status was analyzed by mass spectrometry coupled with liquid chromatograph, and this technique provided a clinical value similar to that of NGF [59]. Impressive results have been obtained using triplet KRd as consolidation as reported by several studies. In the phase II study from the Multiple Myeloma Research Consortium (MMRC) including 76 patients, sCR after eight cycles of KRd (four as induction therapy and four as consolidation after ASCT) was 60%, with 52% of patients obtaining MRD negativity. After a median follow-up of 56 months, median PFS and OS were not reached, being 72% and 84%, respectively, at five years; in MRD-negative patients, these measures were 85% and 91%, respectively [31]. Similarly, a phase II study by IFM reported an sCR rate of 62% after consolidation with four KRd cycles and MRD negativity of 92.6% and 63% at levels of 2.5 × 10^−5^ and 10^−6^, respectively. After a median follow-up comparable to that of the MMRC study (60.5 months), 5-year PFS was 45.1% in all populations, being about 60% in MRD-negative and 35% in MRD-positive patients [32]. In the randomized phase II FORTE trial consolidation, four KRd cycles were found to be more effective than a consolidation with KCd in terms of quality of response (at least CR documented in 54% and 42% of patients receiving KRd and KCd after consolidation, respectively), MRD negativity (62% vs. 43%), and 1-year sustained MRD negativity (47% vs. 25%) [19]. Quadruplet regimens including daratumumab such as D-VTd and D-VRd (two cycles) administered after ASCT were able to improve responses in the CASSIOPEIA and GRIFFIN studies. In the former study, in patients achieving at least CR after consolidation, the MRD negativity rate (33.7% vs. 19.9%, *p* < 0.0001) and ≥ one year sustained MRD negativity rate (50.1% vs. 30.1%, *p* < 0.0001) were significantly higher with D-VTd compared to VTd induction/consolidation [60]. In the GRIFFIN study, MRD negativity at level of 10^−5^ increased from 22% after induction to 50% after consolidation, and sustained MRD negativity lasting at least 12 months was significantly higher in the D-VRd arm than in the VRd one (44.2% vs. 12.6%, *p* < 0.0001), translating into a 3-year PFS of 88.9% (vs. 81.2% for VRd) [61]. 

Few prospective studies addressed the effect of consolidation treatment in the field of ASCT approach. The EMN02/HOVON95 trial, including 1503 patients, compared two cycles with VRD or no consolidation after intensification with ASCT (single or double) or bortezomib, melphalan or prednisone (VMP). After a median follow-up of 74.8 months, consolidation was associated with a significantly prolonged PFS (median 59.3 months vs. 42.9 months, HR = 0.81, *p* = 0.016) with OS not reached in both arms despite OS curve separation after 5–6 years in favor of consolidation [62]. Remarkably, in patients with MRD negativity after consolidation, median PFS was 87 months vs. 38 months in MRD positive (HR = 0.39, *p* < 0.001), whereas 5-year OS was 82% vs. 69%, respectively (HR = 0.51, *p* = 0.01) [63]. On the other hand, the phase III BMT CTN00702 STaMINA trial failed to demonstrate a benefit of consolidation therapy [64]. Within 12 months from starting induction, 758 patients were randomized to receive double ASCT plus lenalidomide maintenance, ASCT followed by four cycles of VRD as consolidation and lenalidomide maintenance, and single ASCT plus lenalidomide maintenance [64]. After a median follow-up of 76 months, respective 5-year PFS was 47.5%, 44.1%, and 45%, (*p* = 0.685) with no differences regarding OS among study arms (5-year 74.7%, 75.4%, and 76.4%, respectively; *p* = 0.745) [65]. However, it has to be noted that in the StaMINA study, induction therapy could last up to 12 months, and most patients received VRD regimes. In contrast to the StaMINA trial, showing no benefit of double ASCT, in the EMN02/HOVON95 trial, double ASCT significantly improved 5-year PFS (53.5% with double vs. 44.9% with single, HR = 0.74, *p* = 0.036) and 5-year OS (80.3% vs. 72.6%, respectively) [18]. Based on these conflicting results, the most recent ESMO guidelines do not recommend consolidation therapy, including double ASCT, as standard therapy post ASCT in all patients [8].

## 5. A Look at ASCT in the Era of Novel Drugs, New Regimens, and CAR-T Cell Therapies

The availability of regimens inducing high MRD negativity rates after induction/consolidation therapies and, particularly, sustained MRD negativity in many patients led to questioning the role and timing of ASCT in MM patients. A phase III study conducted in Italy demonstrated the superiority of double ASCT compared with MPR (melphalan, prednisone, lenalidomide) for six cycles after an induction therapy with four cycles of lenalidomide plus dexamethasone (Rd) in all patients. PFS was significantly longer in patients who received ASCT (median 43 months vs. 22.4 months, HR = 0.44, *p* < 0.001) as well as OS (4-year 81.6% vs. 65.3%, HR = 0.55, *p* = 0.02) [15]. In another similar phase III trial by EMN, patients received four cycles of Rd as induction, after which they were randomized to receive either six cycles of cyclophosphamide, lenalidomide, and dexamethasone (CRd) or ASCT. In this study, PFS (median 42 months vs. 28 months, HR = 0.67, *p* = 0.014) and OS (4-year 87% vs. 71%, HR = 0.51, *p* = 0.028) were significantly better with ASCT than with CRd [16]. These results might be explained with the use, in both trials, of regimens (Rd, MPR, CRd) not as effective as those currently administered as induction/consolidation. However, in patients who received an induction therapy with three cycles of VRD and thereafter a consolidation with five cycles of VRD or with ASCT followed by two additional cycles of VRD, the phase III IFM 2009 study confirmed the benefit of ASCT in terms of PFS, being 47.3 months in the ASCT group vs. 35 months in the VRD group (HR = 0.70, *p* < 0.001), after a median follow-up of 93 months. Median OS was not reached in either group, and it was 62.2% and 60.2%, respectively, at eight years (HR = 1.03, *p* = 0.81) [17,66]. In the EMN02/HOVON95 study, median PFS was significantly improved with ASCT compared with VMP (56.7 months vs. 41.9 months, HR = 0.73, *p* = 0.0001) but no difference was found in OS, i.e., 75.1% and 71,6%, respectively, at five years, after a median follow-up of 60.5 months [18]. The superiority of an intensification with ASCT compared with carfilzomib-based regimens has been recently demonstrated in the UNITO-MM-01/FORTE study in which 474 patients were randomly assigned to four cycles of KRd as induction followed by ASCT and four cycles of KRd as consolidation and 12 cycles of KRd or four cycles of KCd (carfilzomib, cyclophosphamide, dexamethasone)–ASCT–four cycles of KCd. After a median follow-up of 50.9 months, median PFS was not reached in the KRd plus ASCT arm, with 55.3 months in the KRd12 arm and 53 months in the KCd plus ASCT arm, with patients in the KRd-ASCT group having a significant reduction in the risk of progression or death compared with the KRd12 group (HR = 0.61, *p* = 0.0084). However, no significant differences in terms of PFS between KRd12 and KCd-ASCT were reported. Remarkably, in patients with MRD negativity after consolidation, 4-year PFS was 83%, 69%, and 63% in the KRd-ASCT, KRd12, and KCd-ASCT arms, respectively, whereas PFS was similar in the three groups in patients who achieved a 1-year sustained MRD negativity [19]. The phase II randomized CARDAMON trial has explored four courses of KCd as induction followed by ASCT or intensification with four cycles KCd. After a median follow-up of 32.1 months, KCd consolidation did not meet the statistical boundary for non-inferiority since the 2-year PFS, primary endpoint of the study, was 76.1% in patients receiving ASCT vs. 68.6% in those treated with KCd. MRD negativity rates were 47.7% and 30.3% after ASCT and KCd, respectively, with MRD-negative patients having a superior outcome irrespective of treatment arm [67]. In Table 1, we summarized survival measures reported with the more recent clinical trials in the ASCT setting. Until clinical trials show something different, induction followed by ASCT remains the recommended treatment in eligible patients younger than 70 years [8].

The approval of CAR-T cell therapy for MM will probably change the role of ASCT in the near future. Idecabtagene vicleucel (Ide-Cel) was the first anti-BCMA CAR-T cell therapy approved by the Food and Drug Administration (FDA) for RRMM with ≥ three lines of previous therapies, and it is now experimented for NDMM. The phase I KarMMa-4 trial is enrolling high-risk NDMM patients who, after ≤ 3 cycles of an induction regimen, receive CAR-T cells as a consolidation strategy. Ciltacabtagene autoleucel (Cilta-Cl) is another anti-BCMA CAR-T product not yet approved. It was demonstrated to induce an ORR of about 98% in a very heavily pre-treated RRMM population included in the CARTITUDE-1 trial [68]. The phase III CARTITUDE-5 trial is enrolling NDMM patients for whom ASCT is not planned as initial therapy, randomizing them between VRd-Rd and VRd followed by Cilta-Celinfusion, with PFS as the primary endpoint. Tandem ASCT with anti-CD19 and anti-BCMA CAR T-cell infusion is studied in the phase 1/2 SZ-CART-MM02 study in high-risk NDMM. The authors hypothesize that the immune system may be remodeled following ASCT, which may contribute to a higher expansion of CAR T-cells. Enrollment is ongoing. Considering the promising results in the RRMM setting and waiting for results of the above-mentioned trials, whether CART cell therapy may replace the role of ASCT as a consolidation strategy in NDMM, in particular for high-risk patients, remains an open question. 

## 6. Where We Are with Maintenance Therapy

After several phase III trials such as IFM-2005-02 [69], GIMEMA RV-MM-PI-209 [15], and CALGB 100,104, [70] reported a significantly longer PFS in patients receiving lenalidomide maintenance vs. no maintenance after ASCT; the meta-analysis by Mc Carthy et al. including 1208 enrolled in the three trials confirmed the benefit in terms of PFS with lenalidomide and demonstrated a significant improvement in OS [71], seen only in the CALGB study [70]. After a median follow-up of 79.5 months, median PFS was 52.8 months for the lenalidomide group and 23.5 months for the placebo/observational group (HR = 0.48) whereas median OS was not reached and was 86 months, respectively (HR = 0.75, *p* = 0.001) [71]. In the transplant-eligible pathway of the Myeloma XI trial, lenalidomide maintenance significantly improved the median PFS, being 30 months in the observational arm and 57 months in lenalidomide arm (HR = 0.48, *p* < 0.0001), and 3-year OS was 80.2% with observation and 87.5% with lenalidomide (HR = 0.69, *p* = 0.14) [72]. Maintenance with lenalidomide is considered the standard of care in all patients who undergo ASCT [8] but the issue regarding the duration of maintenance remains a hot topic due to different lengths of maintenance therapy in the four phase III trials. CALGB, GIMEMA, and Myeloma XI studies planned lenalidomide maintenance until progression or intolerance while in the IFM trial, lenalidomide was discontinued after a median of two years due to an increased incidence of secondary primary malignancies (SPMs). GMMG-MM5 is the first study to evaluate the optimum duration of lenalidomide maintenance since, after an induction therapy followed by single or double ASCT, patients were randomized to receive lenalidomide maintenance for two years vs. lenalidomide until CR if not obtained previously. After a median follow-up of 60 months, PFS did not differ between the two strategies (3-year PFS = 56% vs. 49.4%, respectively) whereas OS was significantly prolonged in the group receiving lenalidomide for two years (3-year OS = 84% vs. 76%, HR = 1.42, *p* = 0.003) [73]. The comparison between results from the IFM 2009 study [66], in which all patients received lenalidomide maintenance for one year, and those from the collaborative parallel US DETERMINATION trial, planning a lenalidomide maintenance until progression disease, will probably clarify this crucial issue. However, the criterion chosen to discontinue lenalidomide maintenance in the GMMG-MM study was the achievement of CR, but we know that MRD negativity is a better predictor of PFS and OS than CR [56], and several studies demonstrated that lenalidomide maintenance is able to affect MRD status. The median PFS (not reached vs. 20 months, *p* < 0.001) and 3-year OS (96% vs. 86%, *p* = 0.008) after completion of one year of lenalidomide maintenance were significantly better in MRD negative vs. MRD positive patients in the IFM 2009 study [66]. In patients enrolled in the EMN02/HOVON95 trial, lenalidomide maintenance improved MRD negativity by 41%, and patients with sustained MRD negativity (one year) had a superior PFS and OS in comparison with patients with persistent positivity [63]. Finally, in the Myeloma XI study achieving MRD negative status at six months, post lenalidomide maintenance was associated with an 80% reduction in the risk of PFS events (HR = 0.20) and a 67% reduction in the risk of death (HR = 0.33) [74]. A prospective study done at the Memorial Sloan Kettering Cancer Center (MSKCC) assessing longitudinal MRD status (dynamics) in patients receiving lenalidomide maintenance post ASCT showed that patients who lost MRD negativity at two years were more likely to progress than those with sustained MRD negativity (HR Inf, *p* < 0.0001) and those with persistent MRD positivity (HR = 5.88, *p* = 0.015) [75]. 

With regards to proteasome inhibitors as maintenance therapy, bortezomib has been found to improve PFS (but not OS) compared with thalidomide in the HOVON-65/GMMG-HD4 trial, but induction therapy was different between the two groups of patients, with the thalidomide maintenance group treated with vincristine, doxorubicin, and dexamethasone (VAD) and the bortezomib maintenance group receiving bortezomib, doxorubicin, and dexamethasone (PAD) [76]. No prospective study has compared lenalidomide with bortezomib in the post ASCT setting, but in a retrospective analysis of GMMG trials, no difference was found in terms of OS between lenalidomide and bortezomib administered for two years while a significant PFS benefit was observed for lenalidomide maintenance after eliminating the impact of different double ASCT rates [77]. Oral ixazomib was explored as maintenance therapy post ASCT in the phase III TOURMALINE-MM3 study, showing a 39% improvement in PFS with ixazomib vs. placebo (median 26.5 months vs. 21.3 months, HR = 0.72, *p* = 0.0023) [78]. However, bortezomib and ixazomib have not been approved by EMA for maintenance post ASCT. Several studies have assessed drug combinations as maintenance therapy after ASCT starting from lenalidomide, dexamethasone, and ixazomib (IRd) compared with Rd administered for two years in the GEM12MENOS65 trial. After a median follow-up of 56 months, adding ixazomib to Rd did not add benefit in terms of PFS [79]. On the contrary, carfilzomib combined with lenalidomide (KR) as maintenance resulted in improved PFS compared with lenalidomide (R) in the FORTE trial in which 3-year PFS was 75% vs. 65% in the KR and R group, respectively (HR = 0.64, *p* = 0.023) with no differences in terms of OS [19]. CASSIOPEIA is the first study showing a clinical benefit of daratumumab maintenance compared with observation after ASCT [80]. In patients receiving daratumumab for up to two years after consolidation median PFS was not reached vs. 46.7 months with observation (HR = 0.53, *p* < 0.0001). However, while a PFS benefit was seen in the VTd induction/consolidation followed by daratumumab maintenance compared with VTd plus observation only (HR = 0.32, *p* < 0.0001), no difference in terms of PFS was observed between D-VTd induction/consolidation plus daratumumab vs. D-VTd plus observation (HR = 1.02, *p* = 0.91). One-year sustained MRD negativity at a level of 10^−5^ in patients achieving at least CR was 42.3% in patients receiving daratumumab maintenance vs. 31.5% in observation group (OR = 1.71, *p* = 0.0004), with the highest sustained MRD negativity observed in patients receiving daratumumab maintenance after D-VTd (48% vs. 36% in VTd followed by daratumumab group) [60]. Daratumumab plus lenalidomide (DR) after consolidation with D-VRd has been compared with lenalidomide maintenance after VRd consolidation in the phase II GRIFFIN study [34]. After two years of maintenance therapy, 82% of patients receiving D-VRd-DR obtained CR or better, compared with 60.8% in the VRd-R group (*p* = 0.0096), and MRD negativity at 10^−5^ was 64.4% and 30.1%, respectively (*p* < 0.0001). After a median follow-up of 38.6 months, 3-year PFS was 88.9% for D-VRd and 81.2% for VRd [61]. Other ongoing phase III trials such as PERSEUS, AURIGA, and DRAMMATIC are comparing DR vs. R as maintenance after ASCT, whereas EMN18 is exploring ixazomib alone or in combination with daratumumab. All these studies will shed light on the role of daratumumab in the maintenance setting.

## 7. The Role of ASCT in High-Risk Patients

Despite recent advances in upfront therapy in MM, 15–20% of all patients have a predicted OS less than three years. This group of patients can be defined as high-risk MM patients (HR). The correct definition of “high-risk MM” is very difficult in the modern era, when not only stage ISS, cytogenetic abnormalities [t(4;14), t(14;16), t(14;20), copy number abnormalities such as gain(1q) and del(17p)], elevated LDH value, renal failure, the presence of extramedullary disease or plasma cell leukemia are considered markers of HR MM. Now it could be necessary to evaluate other novel aspects such as gene expression profiling, somatic mutations at diagnosis, chromothripsis, TP53 biallelic inactivation, and some dynamic characteristics such as deepness of response to therapy through MRD assessment (in bone marrow samples, imaging PET-CT or circulating plasma cells) in order to build a dynamic risk assessment [81,82]. Indeed, patients experiencing relapse within 18 months of diagnosis are considered to have functional HR MM. Unfortunately, randomized trials have not risk-stratified patients at study entry, but we have only data moving from high-risk subgroups in either planned or post-hoc analyses [83]. Cavo et al. demonstrated the benefit with double HSCT compared to single HSCT in the ITT population of the EMN02/H095 randomized, phase III clinical trial. In particular, the magnitude of this benefit was higher for patients with HR cytogenetics [HR 0.24 for progression, HR 0.30 for death in the subgroup of patients with del(17p)] [18]. Indeed, according to the most recent ESMO recommendations [8], double HSCT can be considered an upfront consolidation strategy for high-risk myeloma (e.g., patients with a del17p cytogenetic abnormality). While the role of double HSCT seemed to be quite established in HR MM, data are lacking about the actual role of upfront single HSCT compared to treatments with novel agents in the absence of HSCT. A subgroup analysis from the phase III ENDURANCE (ECOG-ACRIN E1A11) trial demonstrated inferior PFS in patients with chromosome 1 abnormalities (gain 1q, amp 1q, and del 1p) compared to patients with standard-risk (SR) cytogenetics treated with standard triplet induction regimens without HSCT. Enrolled patients were not intended for ASCT and were randomized to receive KRd vs. VRd induction treatment, followed by progression or 2-year lenalidomide maintenance. Despite that it was a post-hoc analysis, KRd seemed to overcome the negative OS impact among patients with gain 1q and del 1p, but not among those with amp 1q [84]. In the recently published FORTE trial, the benefit of KRd and transplantation over KRd for 12 cycles or KCd plus ASCT was retained in SR, HR, and double-hit (DH) patients defined by the presence of ≥ two chromosomal abnormalities. In SR patients, 4-year PFS was 82% vs. 67% in KRd-ASCT and KRd12 groups, respectively (HR = 0.43, *p* = 0.032); in HR patients, it was 62% vs. 45% (HR = 0.61, *p* = 0.040), and in the DH group, 55% vs. 33% (HR = 0.52, *p* = 0.063), respectively [85]. In contrast, similar PFS rates with KCd plus ASCT and KCd induction/consolidation were seen within the high-risk subset of the phase III CARDAMOM trial, and these results may suggest that ASCT exerts lower efficacy in the high-risk patients when triplet combination used as induction therapy does not include a PI and an IMID [86]. 

Despite a limited number of patients, adding daratumumab to VRd seemed to improve outcomes in the HR group of the GRIFFIN trial; median PFS was not reached in HR patients receiving D-VRd vs. 36 months in those treated with VRd after a median follow-up of 38.6 months [87]. Giving the superiority of KRd in HR MM, the step forward has been to add monoclonal antibodies to this triplet combination. In the phase II MASTER study, SR and HR patients achieved similar rates of MRD negativity and had a comparable risk of MRD resurgence or progression when treated with D-KRd followed by ASCT and an MRD-adapted consolidation/maintenance therapy. Remarkably, based on an ultrasensitive quantitative MRD assay using NGS (10^−6^ level), the greatest impact of ASCT was seen in the ultra-high risk group, defined by the presence of two or more high-risk abnormalities as t(4;14), t(14;16), del(17p), gain or amplification of 1q and t(14;20). In these patients, MRD negativity was 10% before ASCT and 43% after ASCT, showing the significant effectiveness of ASCT in the context of quadruplet induction regimens [88]. High-risk MM patients achieving undetectable MRD after VRD induction/consolidation had a similar outcome to those with SR disease in the PETHEMA/GEM2012MENOS65 study. Moreover, using NGF to isolate MRD followed by whole-exome sequencing of paired diagnostic and MRD tumor cells, it was demonstrated that there was greater clonal selection in patients with SR MM, with higher genomic instability with the acquisition of new mutations in HR patients with no specific abnormalities driving MRD resistance [89].

SWOG-1211, the first randomized study including 100 HR MM patients, did not meet primary endpoint since no difference in PFS was observed between patients receiving eight cycles of VRd regimen vs. eight cycles of VRd plus elotuzumab (median PFS 33.6 months vs. 31.4 months, respectively; HR = 0.968, *p* = 0.45) [90]. Much more encouraging are the preliminary results from the GMMG-CONCEPT trial in which HR patients (defined by t(4;14), t(14;16), del(17p), > 3 copies 1q21 and ISS stage 2 or 3) eligible for ASCT received six cycles with isatuximab plus KRd as induction (I-KRd), four cycles as consolidation after ASCT, and I-KR as maintenance. Preliminary data showed a rapid and deep response rate with 100% of patients obtaining at least PR, 90% at least VGPR, and 46% CR or better after induction, with a 2-year PFS of 75.5% [91]. Kaiser et al. recently reported results of the UK OPTIMUM/MUK nine trial that enrolled 107 ultra-high-risk patients [≥ two high-risk lesions: t(4;14), t(14;16), t(14;20), gain(1q), del(1p), del(17p) or gene expression SKY92 profiling, or with PCL (circulating plasmablasts > 20%)]. Patients received up to six cycles of Dara-CVRd induction, ASCT augmented with bortezomib, followed by Dara-VRd consolidation for 18 cycles, and Dara-R maintenance. Response rates were high in this difficult-to-treat population, with 90% of patients achieving at least VGPR and 68% CR after consolidation. The MRD negativity rate at a level of 10^−5^ was 41% at the end of induction, increasing to 64% at day 100–120 after ASCT, and 18-month PFS was 81.7% [92]. Gagelmann et al. recently published the results of a retrospective study of 623 newly diagnosed multiple myeloma patients with del(17p) and/or t(4;14) undergoing either upfront single autologous (auto), tandem autologous (auto-auto) or tandem autologous/reduced-intensity allogeneic (auto-allo) stem cell transplantation, from 2000 to 2015 (in the era in which novel agents were not yet used). The authors showed that in t(4;14) patients, auto–auto (HR 0.44; *p* = 0.007) and auto–allo (HR = 0.45; *p* = 0.018) were associated with better PFS. In this subgroup, auto–auto also appeared to improve OS compared with single auto (HR = 0.49; *p* = 0.096). In del(17p) patients, only auto–allo appeared to improve PFS [93].

## 8. The Role of ASCT in Relapsed MM Patients

The role of a second ASCT in patients with MM who relapse is debatable considering the plethora or regimens approved for treatment of these patients. According to the most recent guidelines published in 2015 by the American Society of Blood and Marrow Transplantation, European Society for Blood, and Marrow Transplantation, Blood and Marrow Transplantation Clinical Trials Network (BMT CTN) [94], a second ASCT can be planned in patients with a duration of remission longer than 18 months following up-front ASCT. However, considering the median PFS obtained with an approach including induction regimens with triplets or quadruplets, consolidation, and maintenance therapies, these guidelines seem to be outdated. Similarly, outdated are the two randomized clinical trials comparing a nontransplantation approach to salvage ASCT. In the phase III BS BMT/UKMF Myeloma X study, patients relapsing after a previous ASCT were reinduced with bortezomib, doxorubicin, and dexamethasone and then randomized to receive a second ASCT or weekly oral cyclophosphamide for 12 weeks. Median PFS was 19 months vs. 11 months (HR = 0.45; *p* < 0.0001) in patients receiving ASCT and cyclophosphamide, respectively [95]. In the GMMG phase III trial ReLApsE, MM patients with 1st–3rd relapse were randomized to a reinduction with three cycles Rd, ASCT, and lenalidomide maintenance or to Rd continuously. Median PFS was 20.7 months in the transplant arm and 18.8 in the control arm (HR = 0.87; *p* = 0.34) [96]. Prospective randomized trials comparing salvage ASCT with the best non transplant approach could clarify this issue. 

## 9. Perspectives for Tailored Therapies

The introduction of triplet and quadruplet regimens leading to unprecedented high-quality response rates raised the question not only whether ASCT remains the standard treatment in eligible patients but also whether MRD assessment by NGS and NGF can guide treatment intensification or deintensification to avoid toxicity, preserve quality of life, and help the health care system, considering that new therapies are very expensive. Phase II MASTER, enrolling 123 patients, is the first study to have adjusted the intensity of treatment according to MRD assessment [35,88]. All patients received four cycles of D-KRd induction followed by ASCT; subsequently, on the basis of MRD status evaluated by NGS assay (10^−5^ level) at the end of induction, post ASCT and during each 4-cycle block of D-KRd consolidation, they were given 0, 4 or 8 cycles of consolidation therapy. Patients achieving two consecutive MRD negative assessments transitioned to treatment-free observation. Patients who underwent the two phases of consolidation received lenalidomide maintenance. MRD-guided consolidation resulted in an MRD negativity rate of 80% with no differences among patients with SR (78%), HR (82%), and ultra-high-risk cytogenetics (79%). Considering the entire population, 2-year PFS was 87%, being similar in SR (91%) and HR (97%) patients; similarly, 2-year OS was 94%, being 96% and 100% in SR and HR patients, respectively. In contrast, these survival measures were inferior in the ultra-high risk group, showing 2-year PFS and OS of 58% and 76%, respectively, probably due to higher MRD resurgence after treatment cessation. Several ongoing studies conducted in USA and Europe are evaluating MRD-guided treatment modifications in TE MM patients. In the RADAR (UK-MRA Myeloma XV) study, patients achieving MRD negativity through induction/ASCT undergo de-escalation of post-transplant therapy to evaluate whether it is safe and effective, whereas those who remain MRD-positive after ASCT are randomized to different drug combinations including isatuximab as immunotherapy. In the IFM 2020 MIDAS study, all patients received six cycles with Isa-KRD as induction and subsequently were randomized according to MRD status. Patients with MRD < 10^−5^ by NGS after induction are allocated to receive six cycles of Isa-KRD vs. ASCT followed by two cycles of Isa-KRD as consolidation, whereas patients with MRD > 10^−5^ are randomized to ASCT and consolidation with two cycles of Isa-KRD vs. tandem ASCT. Finally, in the OPTIMUM study by NCI, patients undergo MRD testing after 10–15 months of lenalidomide maintenance, and those who remain MRD positive are randomized to lenalidomide vs. lenalidomide plus ixazomib until progression. Two other trials with MRD-driven maintenance are ongoing: the GEM2014 MAIN study by PETHEMA in which patients are allocated to a maintenance therapy with Rd or ixazomib plus Rd for two years and thereafter, if MRD negative, discontinue therapy, but if MRD positive, continue with Rd for three years; the PERSEUS trial by EMN in which patients allocated to the D-VRd arm receive maintenance with daratumumab plus lenalidomide (D-R) for two years and, subsequently, they continue with D-R until progression in the case of MRD positivity, receiving lenalidomide alone for one year if MRD negative.

## 10. Conclusions

The introduction of 3-drug and, more recently, 4-drug combinations as induction and consolidation therapy after ASCT allowed us to achieve rates of MRD negativity, which was unthinkable until recently, leading to improved PFS and OS. MRD status and sustained MRD negativity have been found to be the best tools to predict the outcome of MM patients and, despite not routinely used in clinical practice, MRD assessment has to be implemented since it probably will be essential in the future management of MM. Several questions such as the optimal time point to assess MRD status remain unsolved, but ongoing trials evaluating MRD-driven therapies, particularly consolidation and maintenance, will clarify this issue. 

Moving to the upfront setting, novel therapeutic approaches such as bispecific antibodies, antibody-drug conjugates, and CAR T cells could induce even better results, so in the next years, the role of ASCT is probably destined to change. Nowadays, data from clinical trials continue to demonstrate a significant benefit of ASCT, and it remains the cornerstone of treatment in eligible patients. Despite improvements, outcome of ultra-high-risk MM remains poor, so innovative therapies beyond quadruplets and ASCT have to be explored for these patients. Finally, the main challenges will be to better understand the biology of MM and to make every possible effort for improving the outcome of this heterogeneous disease by personalized precision therapies. 

## Figures and Tables

**Table 1:** Available results of the main phase II/III clinical trials in ASCT.

Trial	Phase	No Pts	Design	Follow-Up	PFS (Median)	OS	Ref.
GIMEMA-MMY-3006	III	480	VTD vs. TD induction and consolidation + 2 ASCT	124 months	60 vs. 41 months	10-yr 60% vs. 46%	[54]
IFM 2009	III	700	VRD induction/consolidation + ASCT vs. VRD	93 months	47.3 vs. 35 months	NR vs. NR	[66]
MMRC	II	76	KRd induction/consolidation, + ASCT	56 months	NR	NR	[31]
IFM KRd	II	46	KRd induction/consolidation, + ASCT	60.5 months	56.4 months	NR	[32]
FORTE	III	474	KRd induction/consolidation + ASCT vs. KCd induction/consolidation + ASCT vs. KRd12	50.9 months	NR vs. 53 months vs. 55.3 months	KRd + ASCT vs. KRd12 HR = 0.61	[19]
CARDAMON	III	278	KCd induction + ASCT vs. KCd	32.1 months	2-yr 76.1% vs. 68.6%	NA	[67]
CASSIOPEIA	III	1085	D-VTd vs. VTd induction/consolidation + ASCT	18.8 months	At 18 months 93% vs. 85%	NR vs. NR	[33]
MASTER	II	123	D-KRd induction/consolidation + ASCT	23.8 months	2-yr 87%	2-yr 94%	[35]
IFM 2018-01	II	45	D-IxaRD induction/consolidation + ASCT	23.6 months	2-yr 95.2%	NA	[37]

VTD (d): VTD(d): bortezomib, thalidomide, dexamethasone; TD: thalidomide, dexamethasone. VRD: bortezomib, lenalidomide, dexamethasone. KRd: carfilzomib, lenalidomide, dexamethasone; R: lenalidomide. KR: carfilzomib, lenalidomide. KCd: carfilzomib, cyclophosphamide, dexamethasone. D-VTd: daratumumab, bortezomib, thalidomide, dexamethasone; D-KRd: daratumumab, carfilzomib, lenalidomide, dexamethasone; D-IxaRd: daratumumab, ixazomib, lenalidomide, dexamethasone. NR: not reached. NA: not available.

## Data Availability

Not applicable.
